# Comparison of Revascularization and Apexification Using Mineral Trioxide Aggregate in Young Human Immature Nonvital Teeth: A Systematic Review and Meta-Analysis

**DOI:** 10.7759/cureus.88732

**Published:** 2025-07-25

**Authors:** Gayatri Pendse, Rashmi Misra, Lalitagauri Mandke, Heervita Maniar, Ankita Khose, Nidhi Basmatkar

**Affiliations:** 1 Department of Conservative Dentistry and Endodontics, DY Patil University School of Dentistry, Navi Mumbai, IND

**Keywords:** apexification, clinical outcomes, dental trauma, immature teeth, mineral trioxide aggregate apical barrier, mineral trioxide aggregate (mta), pulp revascularization, quantitative assessment, radiographic outcomes, regenerative endodontic treatment

## Abstract

The management of immature nonvital permanent teeth in young patients remains a clinical challenge due to open apices and incomplete root development. Revascularization (RET) and apexification using mineral trioxide aggregate (MTA) are the two most widely used treatment modalities, but their comparative effectiveness remains debated. The present systematic review and meta-analysis compared RET and MTA apexification for treating immature nonvital teeth in patients aged 6-18 years. A comprehensive search was performed across PubMed, Scopus, Web of Science, and other dental databases following the Preferred Reporting Items for Systematic Reviews and Meta-Analyses guidelines. Eight studies published between 2016 and 2023, including three randomized controlled trials and five retrospective studies, met the inclusion criteria. Outcomes assessed included clinical success and failure rates, and radiographic changes in root length, root width, and apical diameter. The pooled analysis included 627 teeth. RET showed a higher but statistically nonsignificant clinical success rate (risk ratio, RR = 1.12; 95% confidence interval, CI: 0.99-1.27) and a lower failure rate with apexification (RR = 0.64; 95% CI: 0.28-1.44). RET resulted in significantly greater increases in root length (SMD = 0.31), root width (SMD = 0.61), and apical diameter reduction (SMD = 0.53). No significant heterogeneity or publication bias was detected. Both RET and MTA apexification are effective in achieving periapical healing in immature nonvital teeth. However, RET offers superior regenerative outcomes in root development. Despite a higher risk of discoloration, RET may be the preferred option when proper case selection and aseptic protocols are ensured.

## Introduction and background

The management of immature permanent teeth with pulp necrosis, often resulting from traumatic injuries or deep carious lesions, represents a major clinical challenge in endodontics. These teeth typically exhibit open apices, thin dentinal walls, and incomplete root development, making them structurally compromised and more susceptible to fracture and long-term failure if inadequately treated [1]. The primary therapeutic goal is not only the elimination of infection but also the facilitation of root maturation to improve the tooth’s long-term structural integrity and functional retention.

Over the years, two principal treatment approaches have emerged for these cases: apexification and revascularization (RET), both often incorporating mineral trioxide aggregate (MTA) as a key material in their protocols [2,3]. Apexification traditionally relied on long-term calcium hydroxide therapy to induce an apical barrier; however, MTA has become the material of choice due to its faster setting time, improved sealing capacity, and enhanced ability to stimulate apical hard tissue formation [4,5]. While MTA apexification effectively allows obturation and clinical healing, it does not promote continued root development, which may leave the tooth structurally compromised in the long term.

In contrast, RET, also known as regenerative endodontic therapy (RET), offers the potential for true tissue regeneration within the canal. It leverages the presence of stem cells from the apical papilla, signaling molecules, and a suitable scaffold to regenerate pulp-like tissue that can support continued root growth and apical closure [3]. The technique has shown promising outcomes, including increased root length and dentinal wall thickness, which translate into improved long-term prognosis. MTA is commonly employed as a coronal barrier due to its biocompatibility and sealing properties.

Despite the increasing popularity of RET, there remains significant variability in reported clinical outcomes, with some studies favoring apexification for its predictability and others highlighting the regenerative potential of RET [2,6]. Furthermore, the literature includes both randomized controlled trials and retrospective cohort studies with varying methodologies, inclusion criteria, follow-up durations, and outcome measures. Previous systematic reviews have either focused solely on one technique or lacked quantitative synthesis comparing both interventions side by side in pediatric populations [2].

Thus, a gap exists in the literature for a focused, comparative synthesis of RET vs. MTA apexification specifically in immature, nonvital permanent teeth among young patients, assessing both clinical success and radiographic indicators of root development. Understanding which approach yields superior long-term outcomes, especially in terms of root elongation, apical closure, and dentinal thickening, can substantially influence treatment planning and patient outcomes in pediatric endodontics.

The present systematic review and meta-analysis aimed to address this gap by evaluating the comparative effectiveness of RET and apexification with MTA in young human immature nonvital teeth. The review sought to provide clear, evidence-based insights into clinical and radiographic outcomes, supporting optimal decision-making in regenerative endodontic practice.

## Review

Methodology

Research Design

The present systematic review was conducted in accordance with the Preferred Reporting Items for Systematic Reviews and Meta-Analyses guidelines [6]. The protocol was registered in the PROSPERO database (reference ID: CRD42023463514).

Research Question

This systematic review was structured according to the Population, Intervention, Comparison, Outcomes, and Study design (PICOS) framework to ensure clinical relevance and methodological clarity. The population of interest comprised young patients, typically between 6 and 18 years of age, presenting with immature permanent teeth diagnosed with pulp necrosis. The intervention evaluated was RET, also known as regenerative endodontic therapy, which utilized MTA as part of the protocol. This was compared against apexification using MTA, a conventional method aimed at inducing apical closure. The outcomes assessed included both clinical parameters, such as success and failure rates, and radiographic indicators, including changes in root length, root width, and apical diameter. Based on this framework, the research question guiding the present review is as follows: What is the comparative effectiveness of RET and apexification using MTA in young human immature nonvital permanent teeth, in terms of clinical and radiographic outcomes?

Search Strategy

A comprehensive electronic literature search was conducted across multiple databases, including PubMed, Scopus, Web of Science, EMBASE, CINAHL, Google Scholar, and specialized dental repositories in the subject of Endodontics. The search was executed using a combination of predefined keywords and Medical Subject Headings terms, including "revascularization", "apexification", "Mineral Trioxide Aggregate", "immature non-vital teeth", and "young patients". Boolean operators and truncation strategies were employed to refine search sensitivity and specificity. Filters were applied to limit the results to studies published in the English language. The search spanned from the inception of each database up to the date of final data collection (May 30, 2025). Additionally, the reference lists of included articles were hand-searched to identify any relevant studies that might have been missed during the electronic search. The sample search string for the PubMed database is presented in the Appendix.

Inclusion and Exclusion Criteria

Studies were selected for inclusion if they met the following criteria: 1) they directly compared RET and apexification using MTA in young, immature nonvital permanent teeth; 2) they involved human subjects, specifically pediatric or adolescent populations; 3) they reported measurable clinical and/or radiographic outcomes, including success rates, periapical healing, root maturation, or tooth survival; 4) they were available in full-text format and published in English; and 5) they were published in peer-reviewed journals. Studies were excluded if they were noncomparative in nature, conducted in vitro or on animal models, or presented as review articles, case reports, editorials, or conference abstracts. The PICOS Framework used in the article selection process is listed in Table 1.

**Table 1:** PICOS framework used in the article selection process MTA: mineral trioxide aggregate; RCTs: randomized controlled trials

PICOS component	Description	Inclusion criteria	Exclusion criteria
Population (P)	Young patients with immature, nonvital permanent teeth	Human studies involving children and adolescents aged 6-18 years with immature permanent teeth diagnosed with pulp necrosis	Studies on adults, animal studies, in vitro studies, and teeth with complete root development
Intervention (I)	Revascularization (regenerative endodontic therapy using MTA)	Use of revascularization protocols involving MTA as part of the treatment	Revascularization performed without MTA or unclear protocol
Comparison (C)	Apexification using	Studies that directly compare apexification using MTA to revascularization	Studies using calcium hydroxide alone, or comparing other techniques not involving MTA
Outcomes (O)	Clinical and radiographic outcomes including success rate, failure rate, root length/width, and apical diameter	Reported outcomes related to periapical healing, root development (length/width), apical closure, and adverse events	Studies without quantifiable outcomes or lacking postoperative radiographic/clinical follow-up
Study design (S)	Comparative studies (RCTs, retrospective cohorts)	Randomized controlled trials, prospective/retrospective comparative cohort studies published in peer-reviewed journals, available in full-text and English language	Case reports, case series, editorials, reviews, conference abstracts, and noncomparative designs

Study Selection

The process of study selection was performed in two phases. Initially, two independent reviewers screened titles and abstracts for potential eligibility based on the defined inclusion criteria. In the second phase, full-text articles of the shortlisted studies were retrieved and assessed for final inclusion. Any disagreements during the selection process were resolved through mutual discussion. If consensus could not be reached, a third reviewer was consulted to make the final decision.

Data Extraction

A standardized data extraction form was developed to ensure consistency across studies. Two reviewers (GP and HM) independently extracted data from each included article. The extracted information included study details (author, year, country), study design, sample size, age range, type of intervention (RET or apexification), materials and techniques used (e.g., MTA formulation, and irrigation protocol), follow-up duration, and clinical and radiographic outcomes (e.g., success rates, root length, width, and apical diameter). Data on adverse events or complications were also recorded when available. Any discrepancies between the two reviewers were resolved through discussion. If consensus could not be reached, a third reviewer (LM) was consulted to adjudicate and reach a final decision.

Assessment of Methodological Quality

The quality of the included studies was assessed based on their study design. For observational studies, the Newcastle-Ottawa Scale (NOS) was utilized to evaluate methodological robustness. This scale assesses three key domains: selection of study groups, comparability of groups, and ascertainment of the exposure or outcome [7]. Studies were awarded a score out of 9, with scores of 7-9 considered high quality, 4-6 indicating moderate quality, and 0-3 denoting low quality or a high risk of bias. For randomized or controlled interventional studies, the Cochrane Risk of Bias 2 (RoB 2) tool was employed. This tool evaluates bias across several domains, including random sequence generation, allocation concealment, blinding of participants and personnel, outcome assessment, completeness of outcome data, and selective reporting [8]. A summary of the risk of bias for each included study was graphically represented using the Review Manager (RevMan) software (version 5.3; The Cochrane Collaboration, London, UK), ensuring a transparent appraisal of evidence reliability.

Data Synthesis

A systematic narrative synthesis was initially undertaken to summarize the key characteristics and outcomes of all included studies (Table 2).

**Table 2:** Summary of extracted outcome measures from included studies SD: standard deviation; RET: revascularization; PCO: pulp canal obliteration

Category	Outcome measure	Type	Description
Clinical outcomes	Clinical success rate	Quantitative	Number/percentage of cases with resolution of signs and symptoms (e.g., pain, swelling, and mobility)
	Clinical failure rate	Quantitative	Number/percentage of cases with persistent symptoms or need for retreatment
	Pain or tenderness on percussion	Qualitative	Reported presence or absence of pain/discomfort during clinical follow-up
	Swelling or sinus tract	Qualitative	Presence of soft tissue swelling or sinus/fistula
	Tooth discoloration	Qualitative	Documented occurrence of discoloration, especially in RET-treated teeth
	Tooth fracture	Qualitative	Reported incidence of structural failure posttreatment
Radiographic outcomes	Increase in root length	Quantitative	Measured in millimeter or using standardized indices (e.g., periapical index); expressed as mean ± SD
	Increase in root width	Quantitative	Canal wall thickening; reported as mean difference or standardized mean difference
	Decrease in apical diameter	Quantitative	Reduction in apical foramen width; expressed in mm or % change
	Apical closure/formation of calcified barrier	Qualitative	Radiographic evidence of apical closure or hard tissue barrier
	Periapical healing	Qualitative	Reduction/resolution of apical radiolucency over follow-up period
Follow-up parameters	Duration of follow-up	Quantitative	Follow-up time in months (range: 1-66 months)
	Time to clinical healing	Quantitative	Interval in months from treatment to symptom resolution
Adverse events	PCO	Qualitative	Noted presence of obliteration of root canal space in RET cases
	Other procedural complications	Qualitative	Includes reinfection, overfilling, or allergic reactions

Where appropriate and where outcome measures were sufficiently homogenous across studies, a meta-analysis was performed to generate a pooled estimate of effect. For dichotomous outcomes, such as clinical success rates and incidence of complications, the risk ratio (RR) with 95% confidence intervals (CIs) was calculated. For continuous data, such as measurements of root development or apical closure, the standardized mean difference (SMD) with 95% CI was used. Meta-analyses were conducted using RevMan, version 5.3. A fixed-effect model was applied in cases where heterogeneity was minimal (defined as p > 0.05 and I² ≤ 24%). In contrast, when heterogeneity was significant, a random-effects model was used to account for between-study variability. Statistical significance was determined at a threshold of p < 0.05.

Heterogeneity across studies was assessed using Cochran’s Q test and the I² statistic, which quantifies the proportion of variation due to heterogeneity rather than chance. As per the Cochrane Handbook, heterogeneity was interpreted as follows: I² values of 0%-40% might not be important, 30%-60% may indicate moderate heterogeneity, 50%-90% may suggest substantial heterogeneity, and values of 75%-100% were considered indicative of considerable heterogeneity. A p value of <0.10 from the Q test was considered to reflect statistically significant heterogeneity. To assess the risk of publication bias, Begg’s funnel plots were generated, plotting effect sizes against their corresponding standard errors. Visual inspection was conducted for any evidence of funnel plot asymmetry, which could suggest potential publication bias or small study effects.

Results

**Figure 1:**
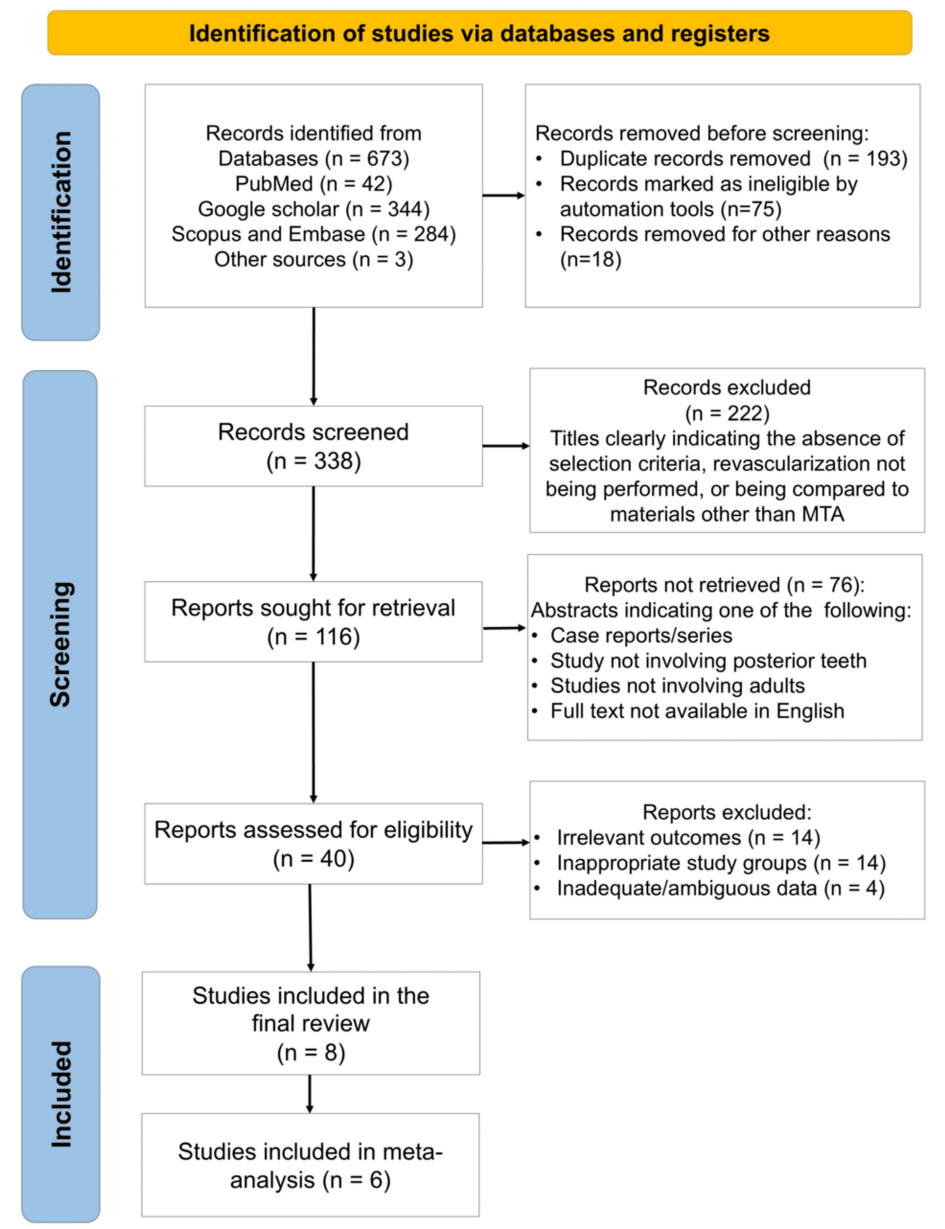
PRISMA flow diagram indicating the selection process of the articles for data synthesis in the present systematic review MTA: mineral trioxide aggregate; PRISMA: Preferred Reporting Items for Systematic reviews and Meta-Analyses

A total of eight studies were identified (Figure 1), which were published between 2016 and 2023 [9-16].

The data extracted from these studies regarding the study designs, methodology, and outcomes are comprehensively summarized in Tables 3-5, respectively. The earliest study comparing RET to MTA apexification was in the year 2016. All the studies were conducted across different countries, including Taiwan, Thailand, Pennsylvania, Egypt, Brazil, Saudi Arabia, Spain, and Pakistan. Of these, an equal number of studies were randomized controlled trials and retrospective studies (n = 4 each, respectively). The sample size across the included studies ranged from 21 to 138. The age range of the samples used in these studies ranged from 6 to 18 years, with a mean age of 10-11 years across all studies.

**Table 3:** Design and characteristics-related data of the studies included in the present systematic review NP: not provided; MTA: mineral trioxide aggregate

Sr. no.	Study (year)	Country	Study design	Sample size	Age	Inclusion criteria	Exclusion criteria	Groups
1	Chen and Chen [9] (2016)	Taiwan	Retrospective study	21	Mean = 10.4 and 10.9	No systemic diseases; pulp necrosis in a premolar caused by dens evaginatus fracturing; radiographic outcomes showing an immature apex accompanied by apical lesions; follow-ups at the clinic that continued for more than one year	Cases that involved retreatment or traditional endodontic treatments	Apexification and regenerative endodontic treatment
2	Silujjai and Linsuwanont [10] (2016)	Thailand	Retrospective study	43	Range = 8-46	Cases with nonvital immature permanent teeth that were treated by either MTA apexification or revascularization; cases with at least a one-year follow-up with adequate preoperative, intraoperative, and postoperative data including radiographs	Patients that could not be contacted for follow-up	Apexification and regenerative endodontic treatment
3	Lin et al. [11] (2017)	Pennsylvania	Randomized clinical trial	118	Range = 8-16 (mean = 10.5)	Patients between 6 and 18 years old; pulp necrosis, defined by a negative response to the temperature test and the electric pulp test; radiographic evidence of immature teeth with a single canal, open apices larger than 1 mm in diameter, and presence of periapical radiolucency; the involved tooth had either dens evaginatus or a history of trauma	Patients with chronic systematic disease; patients allergic to the antibiotics used in the study; vital tooth or tooth with periodontal disease; tooth with more than one canal; radiographic evidence of a root fracture	Apexification and regenerative endodontic treatment
4	Dawoud et al. [12] (2020)	Egypt	Randomized controlled trial	30	Range = 7-9	NP	NP	Apexification and regenerative endodontic treatment
5	Pereira et al. [13] (2020)	Brazil	Retrospective study	44	Range = 6-18	Clinical dental records and periapical radiographs of patients who had at least one traumatized immature permanent tooth with nonvital pulps treated by either apexification or regenerative endodontic procedure; the treatment should be completed and the tooth restored; clinical and radiographic follow-up of at least 12 months	Incomplete clinical dental records; poorly processed periapical radiographs or distorted images	Apexification and regenerative endodontic treatment
6	Al-Habib [14] (2022)	Saudi Arabia	Retrospective study	33	Range = 9-16	Immature permanent teeth with necrotic or inflamed pulp with a preoperative, postoperative, and at least a three-month follow-up radiograph after the treatment, with documented progress notes of clinical signs and symptoms of each endodontic appointment	Missing or incomplete clinical records	Vital pulp therapy, revascularization, and apexification
7	Caleza-Jimenez et al. [15] (2022)	Spain	Randomized clinical trial	18	Range = 7-10 (mean = 8)	Patients without systemic diseases; preoperative radiographs showing an immature apex accompanied by apical lesions; radiographic follow-up during at least six months after the end of treatment	Teeth with vertical fractures; teeth with periodontal conditions; nonrestorable teeth	Apexification and regenerative endodontic treatment
8	Saleem et al. [16] (2023)	Pakistan	Randomized clinical trial	138	Range = 6-18 (mean = 11.41)	Young patient aged 6-18 years, with immature teeth with necrotic pulp and open apex, pulp space not needed for post/core, final restoration, and compliant patients	Patients with a history of trauma with close apex, compromised immune status, e.g., uncontrolled diabetes mellitus, renal failure, immunosuppression, severe asthma, bleeding disorder, grossly carious teeth/unrestorable teeth/badly broken teeth, and eating disorder (anorexia, bulimia, and malnutrition)	Apexification and regenerative endodontic treatment

**Table 4:** Methodology-related data of the studies included in the present systematic review CaOH: calcium hydroxide; MTA: mineral trioxide aggregate; NaOCl: sodium hypochlorite; EDTA: ethylenediaminetetraacetic acid; TAP: triple antibiotic paste; wMTA: white mineral trioxide aggregate; GIC: glass ionomer cement; CBCT: cone beam computed tomography; PAI: periapical index; NP: not provided

Sr. no.	Study (year)	Apexification	Irrigant	Bleeding induction by	Steps	Gap between appointments	Final seal	Scoring
1	Chen and Chen [9] (2016)	CaOH and MTA	2.5% NaOCl	#25k file	1. CaOH regeneration; 2. pulp revascularization; 3. MTA regeneration	-	Coverage of MTA with a depth of 3 mm, followed by composite resin filling	PAI based on Brynolf's [10] reference radiographs representing various stages of apical periodontitis
2	Silujjai and Linsuwanont [10] (2016)	MTA	1.5%-2.5% NaOCl and 17% EDTA	Performed at second visit	1. Irrigation and intracanal medicament TAP; 2. pulp revascularization; 3. MTA application	-	Coverage of MTA with 2-3 mm thickness followed by bonded restoration	PAI: the clinical outcome assessment consisted of success or failure and functional retention
3	Lin et al. [11] (2017)	wMTA	1.5% NaOCl, 0.9% saline, and 17% EDTA	#25 file 3 mm past the foramen in the second visit	1. Irrigation and intracanal medicament TAP; 2. pulp revascularization; 3. collagen membrane; 4. wMTA application; 5. GIC seal	3 weeks	wMTA and composite resin	Root length, root thickness, and apical foramen size were measured using CBCT imaging
4	Dawoud et al. [12] (2020)	MTA	2.5% NaOCl	#15 file 2 mm past the foramen	1. Irrigation and intracanal medicament modified TAP; 2. pulp revascularization; 3. MTA application	3 weeks	NP	-
5	Pereira et al. [13] (2020)	wMTA	6% NaOCl, 2% chlorhexidine, saline solution, and EDTA 17%	Manual K files	1. Irrigation and intracanal medicament TAP + CaOH or chlorhexidine; 2. pulp revascularization; 3. collagen fibers; 4. MTA application	3 weeks	Application with a thickness of approximately 2-3 mm and a coronal seal with temporary sealing and bonded restoration	Root length, root width, root canal width, apical diameter, and apical angle using Apixia Digital Imaging scanner (Apixia Inc., City of Industry, CA) and Image J software (National Institutes of Health, Bethesda, MD)
6	Al-Habib [14] (2022)	MTA	3% NaOCl	NP	1. Irrigation and intracanal medicament CaOH; 2. pulp revascularization; 3. MTA application	NP	NP	The outcome of these treatments was assessed as complete healing, incomplete healing, or failure
7	Caleza-Jimenez et al. [15] (2022)	MTA	1.5%-2.5% NaOCl and 17% EDTA	On the second visit	1. Irrigation and intracanal medicament modified TAP; 2. pulp revascularization; 3. MTA application	NP	MTA with an approximate thickness of 2-3 mm composite resin	PAI (from the study by Ørstavik et al.) categories proposed by Friedman and Mor were modified to interpret the outcomes of treatment: healed (PAI score 1-2, with no signs of reabsorption/calcification); in process of healing (PAI score 3-4, with improvement of the score in the follow-up radiographs and no signs of reabsorption/ calcification); or diseased (PAI score increasing or without change as evidenced from the follow-up radiographs after treatment, or with signs of reabsorption/calcification)
8	Saleem et al. [16] (2023)	MTA	NaOCl	On the second visit	1. Irrigation and intracanal medicament CaOH; 2. pulp revascularization; 3. bony chips or synthetic collagen; 4. MTA application	NP	3-4 mm thick MTA and GIC	Thickening of root wall dentine, root growth, and apex formation in radiograph

**Table 5:** Outcome-related data of the studies included in the present systematic review MTA: mineral trioxide aggregate; RET: regenerative endodontic treatment; VPT: vital pulp therapy; NP: not provided

Sr. no.	Study (year)	Complications	Follow-up	Conclusion
1	Chen and Chen [9] (2016)	The primary complication of apexification was tooth fracture (14.3%). Of the 17 regeneration cases, 17.6% exhibited pulp canal obliteration	1, 3, 6, and 12 months	Thoroughly removing the infection source in the root canal not only eliminates apical lesions in teeth with pulp necrosis and immature roots but also increases the possibility of the regeneration of the pulp-dentin composition. The assessment of the radiographic outcomes indicated that the results of the regeneration treatment were identical to those of the conventional apexification treatment. Regenerative endodontic treatment can become a treatment trend for teeth with pulp necrosis and immature roots
2	Silujjai and Linsuwanont [10] (2016)	Failed MTA apexified teeth (five teeth) consisted of two teeth with vertical root fractures, one tooth with a horizontal root fracture, and two teeth with unrestorable crown fractures. All failed revascularized teeth presented with signs and symptoms of apical periodontitis caused by persistent infection	6 months, 12 months, 2 years, 3 years, 4 years, and 5 years	The success rates of MTA apexification and revascularization were 80.77% and 76.47%, and the functional retention rates were 82.76% and 88.24%, respectively
3	Lin et al. [11] (2017)	External root resorption was found in two cases; in the RET cases with successful outcomes, discoloration and calcification were the two main complications	12 months	90% success rate has been observed in the RET group at the 12-month follow-up, and a 100% survival rate was found in both the RET and apexification groups
4	Dawoud et al. [12] (2020)	NP	3, 6, 9, 12, 15, and 18 months	Revascularization and apexification of teeth with necrotic pulps and incomplete apex formation using MTA material was successful in accomplishing satisfactory clinical findings. Both revascularization and MTA apexification techniques resemble in clinical evaluation criteria related to healing of pain or discomfort, tenderness on percussion, mobility scores, and swelling or fistula
5	Pereira et al. [13] (2020)	The regenerative endodontic procedure group showed more adverse events (10 cases) than the apexification group (one case). Tooth discoloration (90%) was the main adverse event found	12-30 months	Apexification and regenerative endodontic procedures provide satisfactory outcomes with clinical success rates in traumatized immature permanent teeth. Regenerative endodontic procedure and apexification showed similar radiographic outcomes of root development, except for root width
6	Al-Habib [14] (2022)	NP	3 months to 3 years	The outcome of VPT, revascularization, and apexification was fairly high, with healing of periapical periodontitis, absence of signs and symptoms, and root maturation
7	Caleza-Jimenez et al. [15] (2022)	NP	6-66 months	Apexification with the placement of an MTA apical plug and pulp regeneration are reliable treatments for nonvital immature teeth. The radiographic outcomes between the immature teeth subjected to apexification with MTA versus those subjected to revascularization were seen to be comparable. Regenerative endodontic treatment yields a comparatively greater increase in root length and width
8	Saleem et al. [16] (2023)	NP	3 and 6 months	The success rate of pulp revascularization of necrotic teeth was higher compared to apexification with MTA. Although apexification using MTA is a treatment option in patients with open apex and necrotic pulp, pulp revascularization will result in not only closure of the apex but also an increase in radicular dentine thickness and length of the root, which is beneficial for long-term prognosis

The inclusion criteria varied across the studies and showed overlap to different extents. These included cases with young patients aged 6-18 years, characterized by immature teeth with necrotic pulp and open apices, pulp spaces not needed for post/core placement, final restorations, and compliant patients. These non-vital, immature permanent teeth were treated by either MTA apexification or RET. Radiographic outcomes showed an immature apex accompanied by apical lesions. Cases with at least a one-year follow-up had adequate preoperative, intraoperative, and postoperative data, including radiographs, and included pulp necrosis in a premolar caused by dens evaginatus fracturing. The retrospective studies used the criteria of having clinical dental records and periapical radiographs of patients who had at least one traumatized immature permanent tooth with nonvital pulps, treated by either apexification or a regenerative endodontic procedure. The treatment should be completed, the tooth restored, and clinical and radiographic follow-up should be performed for at least 12 months. Likewise, the exclusion criteria also varied and included cases that involved retreatment or traditional endodontic treatments, teeth with vertical fractures, teeth with periodontal conditions, nonrestorable teeth, patients with chronic systemic disease, patients allergic to the antibiotics used in the study, vital teeth or teeth with periodontal disease, and teeth with more than one canal.

All the studies compared MTA apexification with RET. Two studies mentioned the use of white mineral trioxide aggregate (wMTA) for apexification, while one study additionally compared the two materials to calcium hydroxide regeneration. All the studies used sodium hypochlorite for irrigation of the prepared root canal, with concentrations varying from 1.5% to 6% across different studies. Two studies additionally used 17% ethylenediaminetetraacetic acid (EDTA), and Pereira et al. (2020) also used 2% chlorhexidine. The RET procedures were all performed in the second appointment. The bleeding in the canal was induced by means of #15 to #25k files in all the studies. A three-week gap between the first and the subsequent visit was mentioned in three studies. During the final restoration, a 2-3 mm layer of MTA was retained adjacent to the pulp chamber, and the final seal was completed with composite resin.

The scoring and clinical success of the outcome were gauged differently by different authors. The scoring systems used were periapical index, healing status at follow-up visits, radiographic root length, root canal width, apical diameter, apical angle, thickness of root wall dentin, root growth, and apex formation. The follow-up period across the studies ranged from 1 to 66 months, with periods of 3-12 months being common across all studies. Chen and Chen in the year 2016 reported that the primary complication of apexification noted was tooth fracture (14.3%), while pulp canal obliteration (17.6%) was noted with the teeth treated with RET. All failed revascularized teeth presented with signs and symptoms of apical periodontitis caused by persistent infection [10]. Al-Habib et al. (2022) reported that tooth discoloration (90%) was the main adverse event found and that the overall incidence of adverse effects was higher in teeth treated with RET compared to those with MTA apexification.

All authors concluded that RET and apexification of teeth with necrotic pulps and incomplete apex formation, using MTA material, were successful in accomplishing satisfactory clinical findings. Both RET and MTA apexification techniques resemble clinical evaluation criteria related to the healing of pain or discomfort, tenderness on percussion, mobility scores, and swelling or fistula formation. Regenerative endodontic treatment yields a comparatively greater increase in root length and width [15]. Saleem et al. in the year 2023 found that the success rate of pulp RET of necrotic teeth was higher compared to apexification with MTA. The author further added that although apexification using MTA is a treatment option for patients with an open apex and necrotic pulp, pulp RET will result in not only the closure of the apex but also in an increase in radicular dentine thickness and the length of the root, which is beneficial for the long-term prognosis.

Methodological Quality of Included Studies

**Figure 2:**
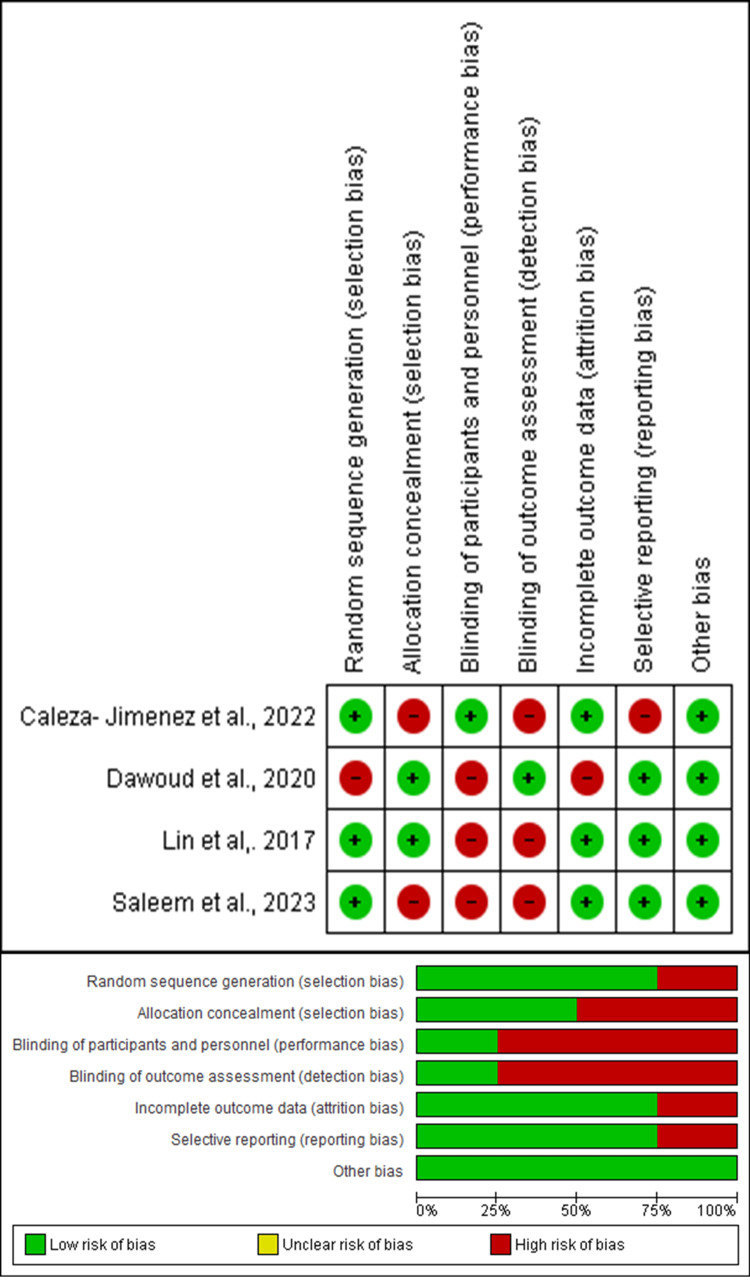
Risk of bias in the clinical studies included in the review Source: [11,12,15,16]

All the included studies were largely comparable in methodological quality. All the included studies had a moderate to high risk of bias in all domains. The highest risk of bias was seen for blinding of participants and personnel (performance bias), followed by blinding of outcome assessment (detection bias) and allocation concealment (selection bias). Among the included studies, all reported the presence of a low to moderate risk of bias. Domains of random sequence generation (selection bias), followed by incomplete outcome data (attrition bias), selective reporting (reporting bias), and other biases, were identified as having the lowest risk of bias by the included studies. The risk of bias in the included studies, as assessed using the Cochrane ROB-2 tool, is depicted in Figure 2.

Among the included studies, the overall quality did not reach the maximum score of the NOS, having the maximum score in the comparability outcome, and was considered to have the highest level of quality with an estimated low risk of bias. The study exhibited a moderate to low risk of bias, with overall quality categorized as good. Risk of bias ratings for the included studies, as assessed by the NOS, are provided in Table 6.

**Table 6:** Risk of bias in the retrospective study through the Newcastle-Ottawa Scale Rating score: ** corresponds to 2, *** corresponds to 3, **** corresponds to 4

Study (year)	Selection (max = 4)	Comparability (max = 2)	Exposure (max = 3)	Overall quality score (max = 9)
Chen and Chen [9] (2016)	**	**	**	6
Silujjai and Linsuwanont [10] (2016)	***	**	***	8
Pereira et al. [13] (2020)	****	**	**	8
Al-Habib et al. [14] (2022)	***	**	**	7

A combined summary table for the risk of bias is listed in Table 7.

**Table 7:** Summary table for overall risk of bias

Study (year)	Study design	Risk of bias tool used	Overall risk of bias
Caleza-Jimenez et al. [15] (2022)	Randomized controlled trial	Cochrane risk of bias 2.0	High
Dawoud et al. [12] (2020)	Randomized controlled trial	Cochrane risk of bias 2.0	High
Lin et al. [11] (2017)	Randomized controlled trial	Cochrane risk of bias 2.0	High
Saleem et al. [16] (2023)	Randomized controlled trial	Cochrane risk of bias 2.0	High
Chen and Chen [9] (2016)	Retrospective study	Newcastle-Ottawa Scale	Moderate
Silujjai and Linsuwanont [10] (2017)	Retrospective study	Newcastle-Ottawa Scale	Low
Pereira et al. [13] (2020)	Retrospective study	Newcastle-Ottawa Scale	Low
Al-Habib et al. [14] (2022)	Retrospective study	Newcastle-Ottawa Scale	Low

Synthesis of results

RR was used to evaluate dichotomous outcomes, while SMD was employed for continuous variables. The meta-analysis assessed the comparative efficacy of RET vs. apexification using MTA across key clinical parameters, including success rate, failure rate, root length and width enhancement, and apical diameter reduction. Each parameter is illustrated through a combined figure that includes both the forest plot and funnel plot for the visual interpretation of effect size and publication bias, respectively.

Success Rate

**Figure 3:**
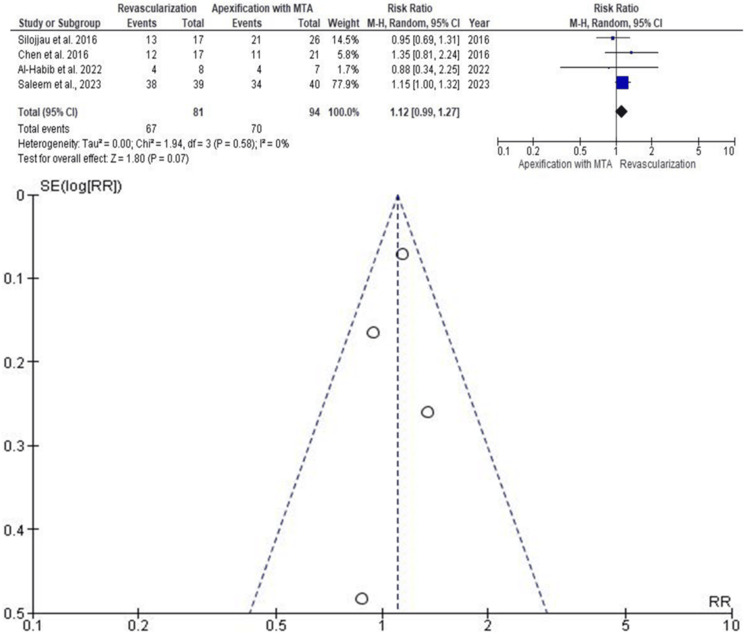
Forest and funnel plots for studies assessing success rate M-H: Mantel-Haenszel; CI: confidence interval; MTA: mineral trioxide aggregate; SE: standard error; RR: risk ratio Source: [9,10,14,16]

Four studies with a pooled sample of 175 teeth (81 RET and 94 apexification) were included to assess healing success (Figure 3). The RR was 1.12 (95% CI: 0.99-1.27), indicating a higher but statistically nonsignificant likelihood of success with RET (p > 0.05). The study by Saleem et al., conducted in the year 2023, contributed the highest weight, while Al-Habib et al. (2022) had the least. The funnel plot revealed no considerable asymmetry, suggesting a low risk of publication bias.

Failure Rate

**Figure 4:**
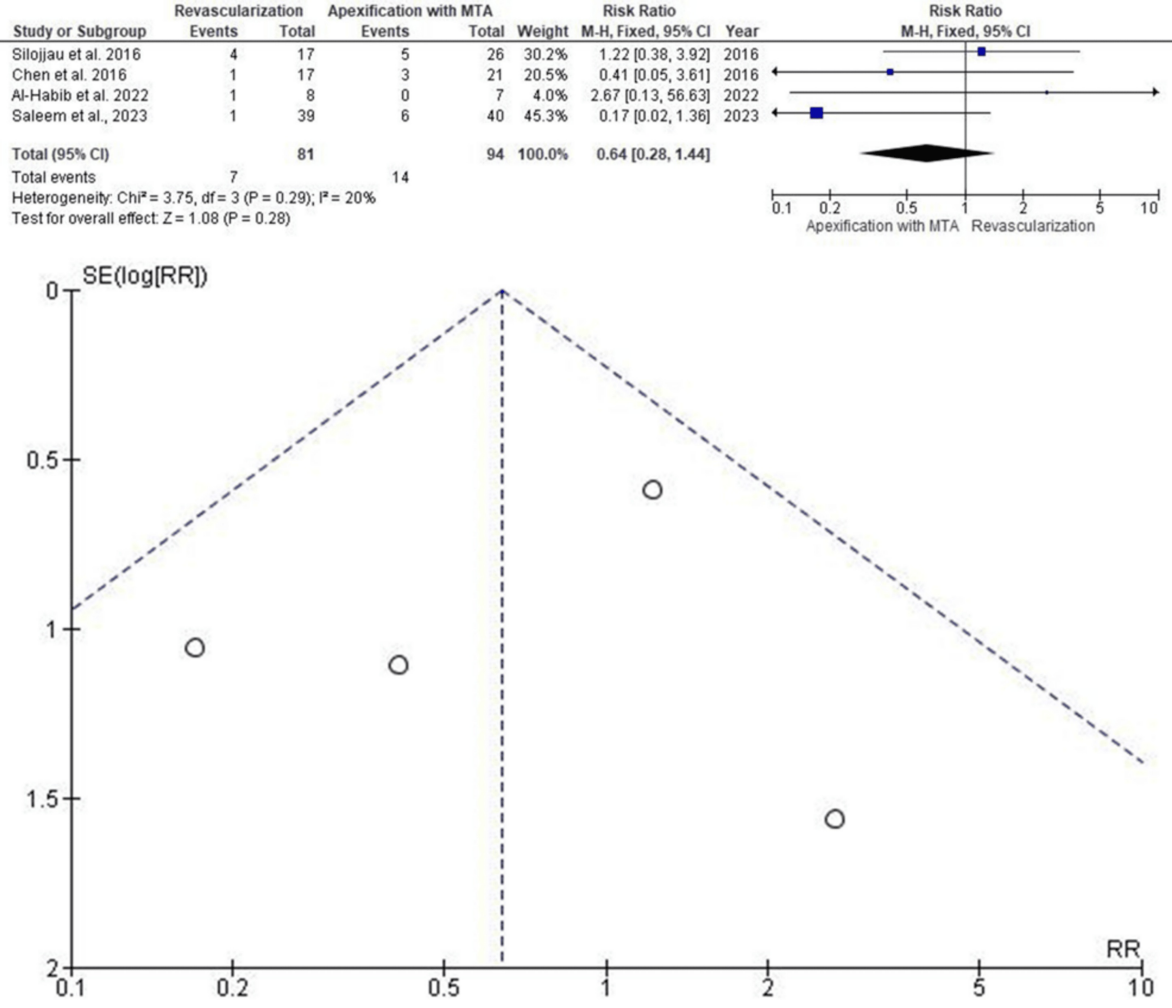
Forest and funnel plots for studies assessing failure rate M-H: Mantel-Haenszel; CI: confidence interval; MTA: mineral trioxide aggregate; SE: standard error; RR: risk ratio Source: [9,10,14,16]

The same four studies also provided data for evaluating treatment failures (Figure 4). The RR was 0.64 (95% CI: 0.28-1.44), with results favoring apexification. Although the point estimate was lower in the apexification group, the difference was not statistically significant (p > 0.05). Again, Saleem et al. (2023) had the greatest influence in the pooled analysis. Absence of funnel plot asymmetry ruled out significant publication bias.

Increase in Root Length

**Figure 5:**
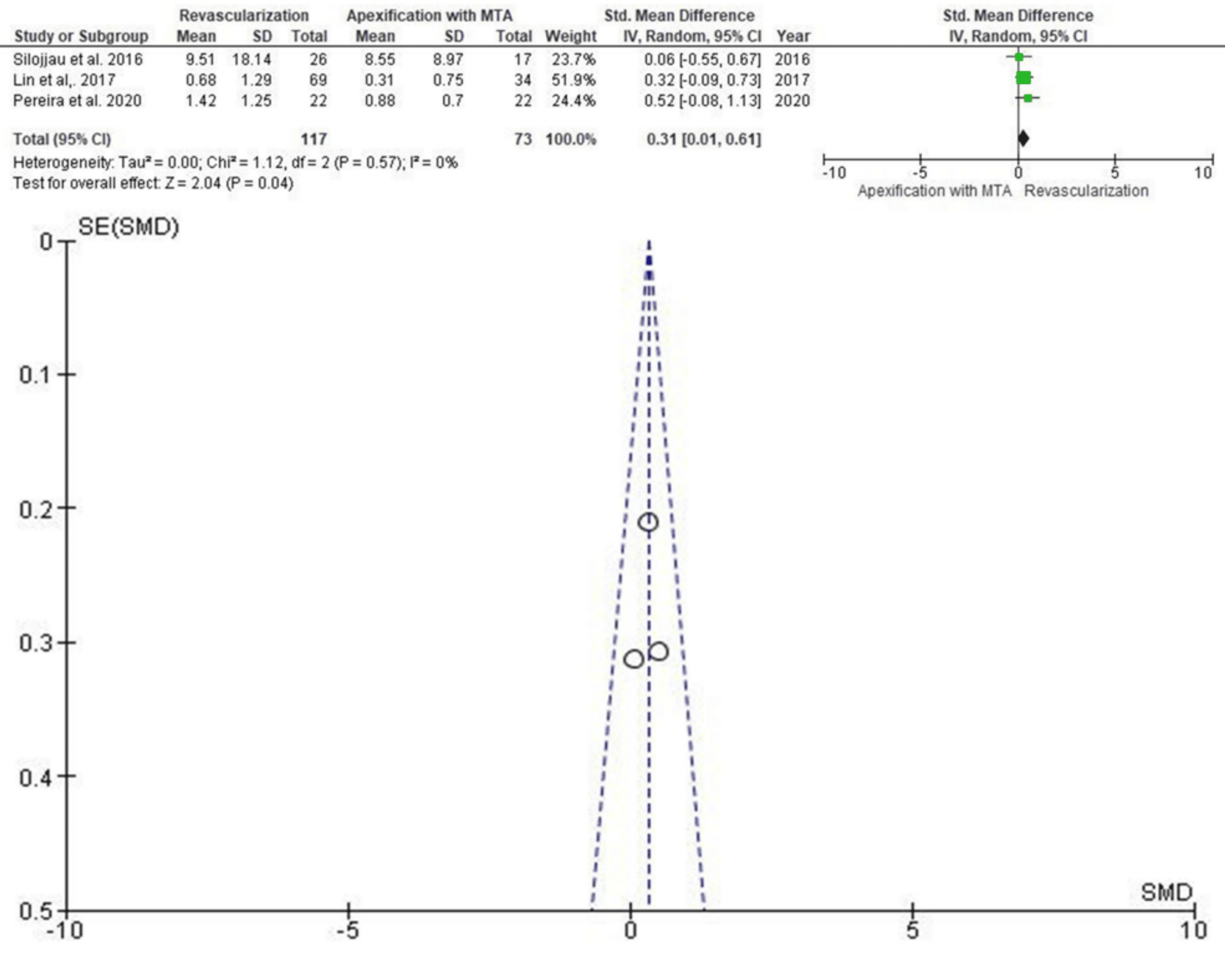
Forest and funnel plot for studies reporting increase in root length SD: standard deviation; IV: intravenous; CI: confidence interval; MTA: mineral trioxide aggregate; SE: standard error; SMD: standardized mean difference Source: [10,11,13]

Three studies involving 190 teeth (117 RET and 73 apexification) evaluated root elongation (Figure 5). The SMD was 0.31 (95% CI: 0.01-0.61), significantly favoring RET (p < 0.05). Lin et al. (2017) held the highest weighting in the analysis. The corresponding funnel plot did not suggest any risk of publication bias.

Increase in Root Width

**Figure 6:**
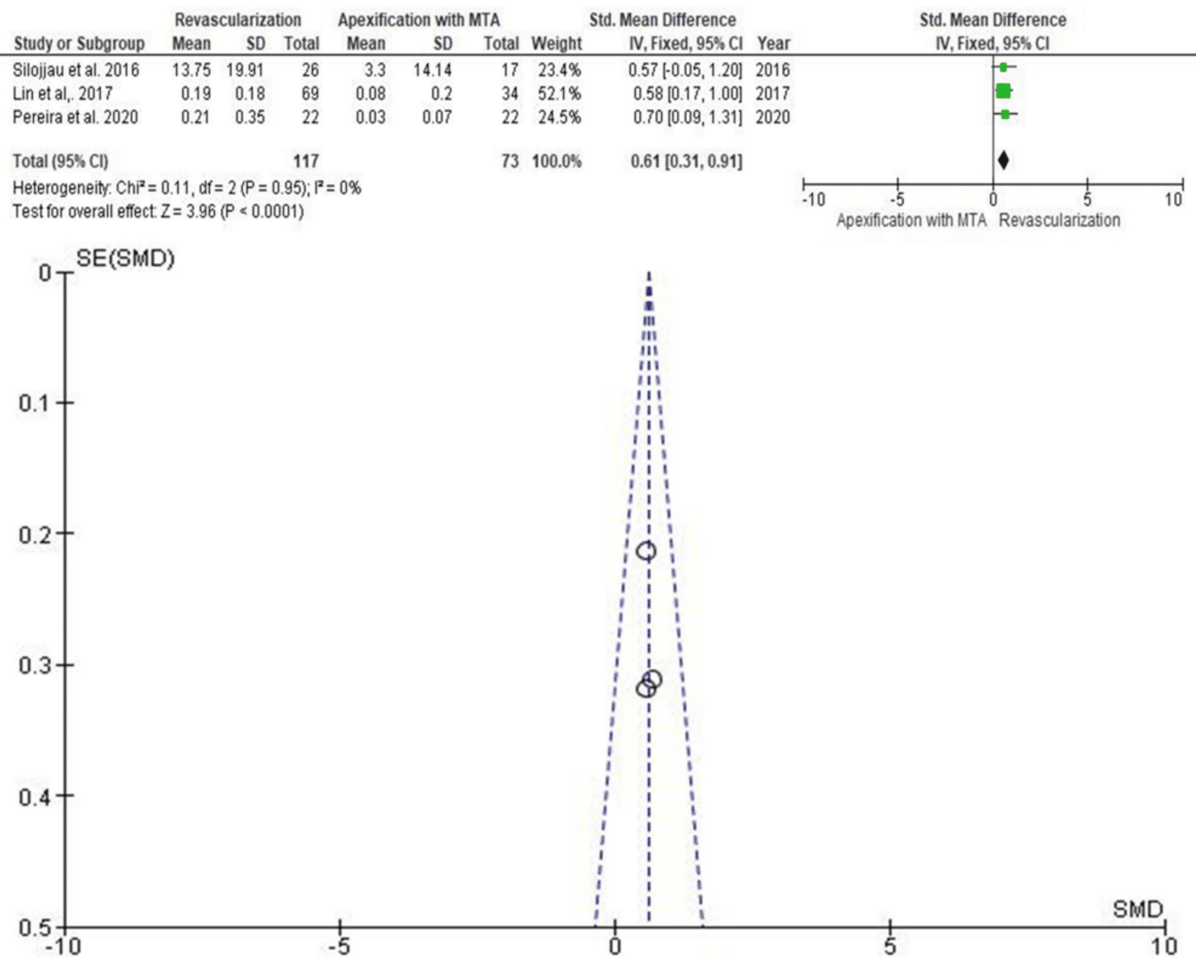
Forest and funnel plot for studies reporting increase in root width SD: standard deviation; IV: intravenous; CI: confidence interval; MTA: mineral trioxide aggregate; SE: standard error; SMD: standardized mean difference Source: [10,11,13]

Root width changes were reported in the same three studies (Figure 6). The pooled SMD was 0.61 (95% CI: 0.31-0.91), with a statistically significant advantage in favor of RET (p < 0.05). Lin et al. (2017) again contributed the highest statistical weight, while Pereira et al. (2020) had the lowest. Funnel plot analysis showed a symmetrical distribution, supporting the absence of reporting bias.

Decrease in Apical Diameter

**Figure 7:**
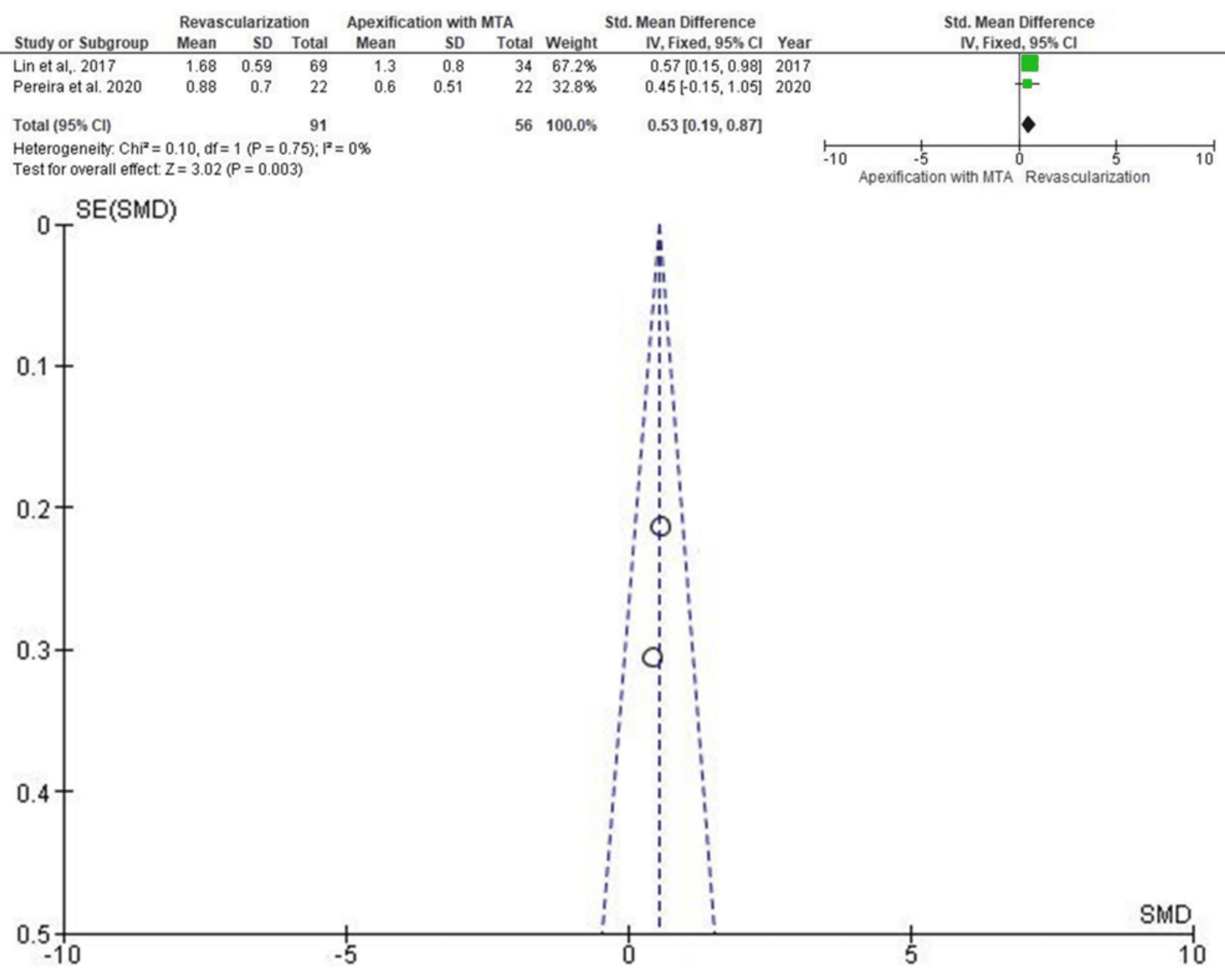
Forest and funnel plot for studies reporting decrease in apical diameter SD: standard deviation; IV: intravenous; CI: confidence interval; MTA: mineral trioxide aggregate; SE: standard error; SMD: standardized mean difference Source: [11,13]

Two studies (147 teeth: 91 RET and 56 apexification) examined changes in apical diameter (Figure 7). The SMD was 0.53 (95% CI: 0.31-0.91), significantly favoring RET (p < 0.05). Lin et al. (2017) again contributed the most weight to the analysis. The funnel plot did not demonstrate any meaningful asymmetry.

Discussion

This systematic review synthesized findings from eight clinical studies published up to 2025, comparing the efficacy of RET and apexification using MTA for managing immature nonvital permanent teeth in pediatric and adolescent populations. The diversity of geographical settings, from Asia and the Middle East to North and South America, demonstrates the global clinical interest in regenerative endodontics. However, the absence of data from India indicates a regional research gap that warrants further investigation [17].

Most studies included either randomized controlled trials or retrospective cohorts, lending a balanced perspective on both controlled interventions and real-world clinical outcomes [18]. The consistent age bracket of 6-18 years reflects a focus on the target demographic for regenerative interventions; however, root development can vary considerably within this range, potentially affecting treatment responses, and should be factored into treatment planning.

While inclusion criteria across studies emphasized necrotic pulps in immature teeth with open apices and apical pathology, heterogeneity in design and patient selection could have influenced outcome variability [19]. Additionally, the application of wMTA in some studies introduced a material variable, with differences in biocompatibility and sealing, potentially impacting the regenerative response. Notably, Pereira et al. (2020) included a comparative arm using calcium hydroxide, providing insights into alternative apexogenesis protocols and underscoring the evolving spectrum of regenerative materials [13]. This diversity in material application adds to the ongoing discussion regarding the most effective agents in promoting apical closure and tissue healing. The exclusion of previously treated teeth or those managed with conventional endodontic approaches reflects an effort to isolate the effects of RET and apexification without confounding from past interventions [20].

Disinfection protocols across studies showed marked variation, particularly in sodium hypochlorite concentrations (ranging from 1.5% to 6%), which could influence clinical outcomes due to its antimicrobial and tissue-dissolving properties [21]. The use of adjunctive irrigants such as 17% EDTA and 2% chlorhexidine, as seen in several studies, reflects clinical efforts to optimize canal conditions for tissue regeneration [22]. A consistent pattern was observed regarding the timing of RET procedures, with a three-week interval between visits being commonly followed to allow clot stabilization and stem cell proliferation [23,24]. Final restorations typically involved placement of 2-3 mm of MTA and composite resin, which aligns with standard RET protocols for effective coronal sealing and structural support.

In terms of outcome assessment, studies utilized objective radiographic parameters, including root length, canal width, and apical diameter. Some studies extended evaluation to dentinal wall thickness and canal morphology, thereby enriching the assessment of regenerative success beyond symptom resolution [25]. Follow-up periods ranged from as short as one month to as long as 66 months, allowing evaluation of both early healing and long-term structural changes [2]. Such variability, however, poses challenges in establishing uniform benchmarks for treatment success.

Complications were common. Chen and Chen (2016) reported root fractures following MTA apexification, highlighting concerns about the structural vulnerability of teeth lacking additional root development [9]. The same study also observed canal obliteration in RET cases, which may complicate future endodontic access [26]. Silujjai and Linsuwanont (2017) associated RET failure with persistent infection, underscoring the necessity of thorough disinfection prior to clot induction [10]. This observation aligns with evidence from Haapasalo et al. (2003), which emphasized the limits of regenerative potential in the presence of unresolved microbial challenge [27]. Esthetic concerns were prominent in Al-Habib’s (2022) study, which reported tooth discoloration in 90% of RET-treated teeth, a significant drawback for anterior applications despite the overall clinical success [14]. Marconyak et al. (2016) further corroborated this, attributing discoloration to MTA and related materials used during RET [28].

From a radiographic perspective, Caleza-Jimenez et al. (2022) found RET to be more effective in promoting root maturation, as evidenced by measurable increases in root length and width [15]. This is particularly important in young patients, where long-term tooth survival depends on continued root development and dentinal reinforcement [29]. Similarly, Saleem et al. (2023) concluded that RET produced superior outcomes in apical closure and radicular dentin apposition when compared to MTA apexification [16], consistent with findings from Bukhari et al. (2016), which also highlighted RET's advantages in regenerative capacity [30].

The quality of included studies significantly influenced the interpretation of results. All four randomized controlled trials were rated as having a high risk of bias, primarily due to lack of blinding and inadequate allocation concealment [11,12,15,16]. These methodological flaws raise concerns about potential performance and detection bias, particularly for subjective outcomes like pain perception or esthetic satisfaction. In contrast, the retrospective studies demonstrated generally low risk of bias according to the NOS, especially in terms of outcome assessment and comparability [10,13,14]. Chen and Chen (2016) was the only retrospective study rated as moderate risk due to weaker selection and exposure reporting [9]. Nevertheless, the consistency of radiographic outcomes across both high- and low-bias studies strengthens confidence in the overall findings favoring RET.

Despite its methodological rigor, this review has several limitations. First, the high risk of bias in all included RCTs limits the strength of the pooled evidence. Second, significant heterogeneity in clinical protocols, including irrigants, medicaments, and follow-up durations, may have impacted outcome comparability. Third, only a limited number of studies reported certain parameters like apical diameter, reducing statistical power for those outcomes. Fourth, all included studies were published in English, introducing potential language bias. Finally, the lack of standardized outcome reporting and variability in radiographic measurement tools may have contributed to over- or underestimation of treatment effects. These limitations highlight the need for future high-quality, multicenter trials using standardized clinical and radiographic criteria.

Overall, both RET and MTA apexification are effective treatment options for immature nonvital teeth; however, RET appears to offer superior potential for continued root development and structural reinforcement. When performed under strict aseptic protocols with appropriate case selection, RET may enhance long-term prognosis, especially in younger patients. Further research should focus on minimizing esthetic complications, refining disinfection protocols, and filling geographical research gaps to support broader clinical adoption.

Clinical recommendations

Based on the current evidence, both RET and apexification using MTA are effective in treating immature nonvital permanent teeth in young patients. However, RET is preferable in cases where long-term structural integrity is a priority, as it promotes continued root development, including significant gains in root length, width, and apical closure. Clinicians should consider RET when the following conditions are met: 1) the patient is young with open apices, 2) the canal can be adequately disinfected, (3) there are no contraindications to bleeding induction, and (4) esthetic concerns regarding potential discoloration can be managed or are of lesser concern (e.g., posterior teeth). Apexification with MTA remains a valid option in cases where RET is contraindicated, patient compliance is poor, or immediate apical barrier formation is clinically desirable. Proper case selection, disinfection protocol adherence, and patient follow-up remain critical to the success of either approach.

## Conclusions

Based on the synthesis of evidence from eight clinical studies, both RET and apexification using MTA are effective in managing immature nonvital permanent teeth, achieving favorable clinical and radiographic outcomes. However, RET demonstrated a comparative advantage in promoting continued root development, including significant increases in root length, width, and apical closure, which are critical for long-term tooth stability and function in young patients. Although apexification remains a reliable option, especially where regenerative protocols are not feasible, RET offers enhanced biological potential, provided strict aseptic protocols and restorative measures are adhered to. The findings support the growing clinical preference for RET in suitable cases, while also emphasizing the need for individualized treatment planning based on patient factors, tooth maturity, and esthetic considerations.

## Appendices

Search string in PubMed

The following search string was used during search: ("revascularization"[MeSH Terms] OR "regenerative endodontic therapy" OR "regenerative endodontics" OR "pulp revascularization") AND ("apexification"[MeSH Terms] OR "mineral trioxide aggregate" OR "MTA apexification") AND ("immature teeth" OR "open apex" OR "non-vital teeth" OR "necrotic pulp") AND ("clinical outcomes" OR "radiographic outcomes" OR "root development") AND ("humans"[MeSH Terms] AND English[lang]).

Filters applied

The following filters were applied during the search: 1) species: humans; 2) language: English; 3) age: child (6-12 years) and adolescent (13-18 years); and 4) publication dates: inception to May 30, 2025.
